# The Difference in Perceptual Anticipation Between Professional Tennis Athletes and Second-Grade Athletes Before Batting

**DOI:** 10.3389/fpsyg.2018.01541

**Published:** 2018-08-22

**Authors:** Rong Shangguan, Yuanyuan Che

**Affiliations:** School of Physical Education, Hunan Normal University, Changsha, China

**Keywords:** tennis professional athletes, second-grade athletes, perceptual anticipation, ERP, P3

## Abstract

To investigate the differences in cognitive processing of perceptual anticipation of tennis players at different levels before batting by the event-related potential (ERP) technique, we used the skilled-expert paradigm. We compared the cognitive and neural mechanisms of professional tennis athletes and second-grade athletes relating to their perceptual anticipation of the batting line at different time points [the time point of the ball landing (T0), and the 80 ms before batting time point (T1)]. The results showed that, regardless of the T0 or T1 time point, professional tennis athletes had shorter anticipation times and higher correct batting line rates than second-grade athletes. The ERP results demonstrated that compared with second-grade athletes, professional tennis athletes induced smaller N1 amplitudes and larger P2 amplitudes in early perceptual anticipation, and induced smaller N2 and larger P3 amplitudes in late perceptual anticipation. These studies suggest that, regardless of whether they are in an early or late stage, tennis professional athletes are faster and more accurate in respect of their perceptual anticipation of tennis lines than second-grade athletes are. This is possible since the relevant neural network of the former is more easily activated and faster. The prefrontal cortex may be a critical area of the brain for perceptual anticipation in tennis.

## Introduction

In our daily lives, it is very important to quickly identify, judge, and act when a situation calls for this response. Especially in sports, it is particularly critical for athletes to judge their opponent’s techniques and tactics and make corresponding changes in their decision-making accordingly. In a game, higher-level athletes will always have a good anticipation of their opponent’s next intention or action, and the stronger an athlete’s professional skills, the faster and more accurate this anticipative ability can be. Poulton (1950) suggests that the cognitive process of predicting or judging future events by using a part of, or knowing about, this motion information is called perceptual anticipation. There is a strong relationship between the basis of perceptual anticipation and the technical level of engagement in a sport.

In search of the advantages gained by high-level athletes using perceptual anticipation, sports psychologists have carried out a lot of research to explain this phenomenon. For example, from the behavioral view, Overney et al. (2008) demonstrated that tennis specialists had a significant speed advantage in perceptual anticipation tasks. Mcpherson (2010) examined the perceptual anticipation ability of female high-level tennis players and found that the anticipation of an expert group about the batting route was significantly more quickly developed than that of a novice group. Moreover, they inferred that there was a set of complex and fine motion images in the brain of tennis experts matching a range of actual situations, and the novice athlete’s lack of this matching cognitive memory structure resulted in significant differences in perceptual anticipation. Huys et al. (2009) and Farrow and Reid (2012) found that the information pickup of skilled athletes was more holistic than that of low-skilled athletes when covering different parts of opponents’ bodies during studies of the anticipation ability of tennis athletes at different professional levels relating to the placement of shot. Loffing et al. (2011) and Smeeton and Huys (2011) studied the ability of high-level tennis players and novice athletes to predict the shot direction of opponents after watching different presentations (such as video presentations, spot presentations). They found that the perceptual anticipation of subjects of both classes on the experimental material with spot presentation is obviously worse than that of the video presentation, but the perceptual anticipation of a high-level tennis player is superior to the novice under various conditions. Therefore, these studies showed that the perceptual anticipation ability of the high-level tennis players mean an advantage over low-level tennis players, but for the time process and space distribution characteristics of this advantage in perceptual anticipation, we still have a poor understanding.

In recent years, a large number of studies have attempted to use event-related potentials (ERPs) to examine the cognitive processes behind the perceptual anticipation of different professional athletes. Vogel and Luck (2000) have shown that ERP components N2 and P3 reflect the psychological components of men related to the subject’s attention and perceptual anticipation. Other studies have found that N2 and P3 are highly correlated with cognitive functions such as the perceptual anticipation in sports (Kok, 2001; Dockree et al., 2005; Kimura et al., 2005). For example, Rose and Christina (1990) found that professional group athletes had an induced earlier and smaller P3 amplitude than novice group players by comparing the perceptual anticipation ability of tennis experts and novices. Nakamoto and Mori (2012) employed the Go/No-Go paradigm to study the neural mechanisms of baseball players performing selection tasks and found that the P3 amplitude induced in the forehead of baseball players in a professional group was significantly higher than that in a novice group. Zhao (2010) explored the cognitive processes of the perceptual anticipation of national Sanda athletes and ordinary students in Sanda elective courses on the professional picturing of different difficulties, and the results were that national team athletes induced an earlier and smaller P3 amplitude than ordinary college students. Xu and Li (2010) studied the characteristics of decision-making in table tennis rotation and found that, compared with ordinary college students professional athletes induced a larger P3 amplitude in the right side of the frontal area and temporal region, and a later P3 amplitude in the right temporoparietal junction. Zhang (2012) used video materials to study the ERP of tennis experts, second-grade, and novice players on the anticipation of batting routes and found that tennis experts showed a significant response-speed advantage before the opponent’s shot of 80 ms ago. In addition, the N1 amplitude induced by experts and second-grade players was significantly smaller than that of the novice, and the expert group players induced the longest latency N2. The novice group induced the longest latency of P3, and the mean amplitude of the later slow wave of the three class players studied was higher at 800–1900 ms in the left hemisphere than that in the right hemisphere. Recently, Wang (2013) used the time-series context paradigm to conduct an ERP study of the anticipation advantages of tennis experts. This study found that the N1 amplitude induced by tennis experts was significantly larger than that of the experienced and novice groups and was critically impacted by the time series in the early stage of the stimulus. In the later stage of the stimulus, the P3 amplitude induced by the professional group of athletes reached its maximum at Pz, and the incubation period was earlier. The results of these studies showed that athletes at higher professional levels had a significant attention advantage in the early stages of perceptual anticipation and showed a finer and more accurate anticipation in the later stages of processing; but the difference between athletes of different levels in the key time nodes of perceptual anticipation remains unclear. Through this review of the literature, we found that both domestic and international sports psychologists have conducted fruitful research on the perceptual anticipation of tennis players, especially with ERP, which helps us to more clearly understand the time course and spatial distribution of perceptual anticipation. However, there are some contradictory themes in our predecessors’ research results, such as differences in ERP amplitude between second-grade athletes (or skilled athletes) and professional athletes, compared to those between second-grade athletes (skilled athletes) and novices. Some researchers have found that professional athletes induced a smaller N1 or P3 amplitude (Rose and Christina, 1990; Zhang, 2012), but others have found that professional athletes induced a larger P3 amplitude (Nakamoto and Mori, 2012; Wang, 2013). What is more, previous studies mostly used game video as the experimental material for perceptual anticipation. These studies had a high ecological nature that could grasp the perceptual anticipation of the athletes overall, but could not study cognitive processing and its temporal and spatial distribution characteristics relating to the perceptual anticipation of athletes at specific time points very finely.

To improve and develop the perceptual anticipation of tennis players, we used ERP technology to let tennis players of different levels anticipate the batting route when watching pictures at different time points. This was to examine the cognitive processing process of tennis players of different levels on the perceptual anticipation of batting routes, and to examine the behavior and cognitive processing differences of professional tennis players and second-grade athletes on perceptual anticipation at different times. We explored the information processing characteristics and distribution principles of cognitive attention of excellent tennis player on the perceptual anticipation of batting routes, and this can provide a theoretical reference for improving the training methods of tennis players.

According to our and others’ previous studies (Zhang, 2012; Shang and Fan, 2015), we can assume that the perceptual anticipation of second-grade athletes and tennis professional athletes at two time points [landed time of ball (T0) and before an opponents’ racket touches the ball 80 ms ago (T1)] exists as differences in ERP characteristics and neural mechanisms. Compared to second-grade athletes, tennis professionals have higher accuracy and faster response time of perceptual anticipation at the T0, T1 time points.

## Materials and Methods

### Subjects

The Expert group was comprised of 15 tennis professional athletes (10 males, 5 females, *M*_age_ = 18.7), with a technical level of 5.5 or more (based on the assessment criteria of the American Tennis Association). The second-grade athletes group was comprised of 15 students of the tennis major in the School of Physical Education at Hunan Normal University (10 males, 5 females, *M*_age_ = 19.7), with a technical level of 3.5 or more. All subjects’ uncorrected vision or corrected visual acuity were normal, all were right handed, in good physical health, and all reported no history of neurological diseases. All subjects received a small gift after completing the experiment.

### Materials

The experimental materials were all from the HD video of the women’s singles at the 2010–2013 French Open Tournaments. According to previous research results (Zhang, 2012; Shang and Fan, 2015), intercepting those pictures where the athlete was on the top of the screen (in video or stalemate stage), we were able to interval freeze a photo every 25 ms from the previous shot, thus each video set was made up of 10 freeze-frame photos. According to our previous study results, at the four key time points of perceptual anticipation [landed time of ball (T0), before opponent’s racket touches the ball 80 ms ago (T1), the moment of batting (T2), and the moment after batting 80 ms later (T3)]; tennis professional athletes and second-grade athletes show differences at the T0 and T1 time points (Shang and Fan, 2015). Therefore, we chose the landed time (T0) and the 80 ms (T1) before batting to explore the differences between the perceptual anticipation of tennis professional athletes and second-grade athletes in the neural mechanism.

#### Material Validity Assessment

We invited 10 people, including the coaches of Hunan and Hubei Province tennis teams, the tennis teachers at the Wuhan Institute of Physical Education and the School of Physical Education of Hunan Normal University, and others to assess the 600 photos in our collection, and finally selected 240 photos as experimental materials. These included, respectively, 60 photos of the landed time of ball (T0), before the opponent’s racket touched the ball 80 ms ago (T1), the moment of batting (T2), and after batting 80 ms later (T3). We also selected 120 photos of the landed time of ball (T0), and before the opponent’s racket touched the ball 80 ms ago (T1).

#### Material Familiarity Assessment

We invited 10 expert and second-grade tennis players to carry out a familiarity assessment on the photos of the landed time of the ball (T0), and before the opponent’s racket touched the ball 80 ms ago (T1). The group used a five-point scale to carry out assessment, and the evaluation results showed that there was no significant difference between the professional tennis player group and the second-grade athletes in the degree of familiarity with the photos, *t*(9) = 1.12, *p*>0.05 (**Table 2**).

The picture material was standardized using Photoshop software and given unified picture resolution, contrast, brightness, and other physical properties. Presentation was random by E-prime2.0 software when in experiment.

### Experimental Design

A three-factor mixed design of 2 (batting route: straight line, oblique line) × 2 (batting time: T0, T1) × 2 (type of subjects: tennis professional athlete and second-grade tennis player) experiment was conducted. In this design, the batting route and batting time are within-subject variables, and type of subject is a between-subject variable. Here, the dependent variable is the accuracy and response time of subjects on the perceptual anticipation of batting routes, and the incubation period and amplitude of the corresponding ERP components (N1, N2, P2, and P3).

### Procedures

**FIGURE 1:**
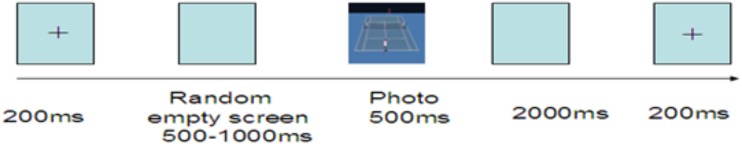
The procedure of single trial.

We presented the experimental material using E-prime 2.0 software (**Figure 1**). Before the experiment, we adjusted the position and sitting position of subjects to keep their eyes 80 cm from the display and at the same level with the center of the display screen. First, the subjects conducted a 10-practice trial, to make the players familiar with the requirements and key responses of the entire experiment.

In the formal experiment, each respondent was first shown “+” for 200 ms, followed by a randomly displayed blank screen for 500–1000 ms, and then shown the stimulating picture for 500 ms, and finally a blank screen for 2000 ms. The task of subjects was to make a fast and good response when the stimulating picture was presented. If the subject thought that the player at the top of the screen would make a straight shot, they were asked to press “1,” and to press “3” if they thought the player would make an oblique line shot. If subjects did not respond in the final blank screen stimulus within 2000 ms, then we gave a warning prompt of “Attention! Do NOT press button!,” and automatically jumped to the next trial. The experiment was divided into two blocks, each block containing 120 trials. The stimulation picture in each trial was randomly presented, and could rest 2 min between blocks.

### EEG Recording

The experiments used NeuroScan ERP as a recording and analysis system. We recorded the EEG in the experiment according to the international standard 10–20 extended 64-bit system of the conductive EEG cap. The tie line of the left mastoid process was used as the reference electrode for online recording. After the experiment, the bilateral mastoid process was transformed into the reference electrode, and we placed HEOG and VEOG on the lateral, and upper and lower sides of the eyes. The filter band is usually 0.05–70 Hz, the sampling frequency 500 Hz/conduct, and scalp resistance < 5 kΩ.

### ERP Processing and Statistics

After recording the consecutive EEG of subjects, we used an offline processing method to calibrate the VEOG in the NeuroScan software and excluded all the artifacts in the experiment. This study only collected and analyzed the EEG data after display of the stimulating message, and overlaid the EEG of individuals stimulated by different pictures. We took an amplitude larger than ± 80 μV as an artifact to be automatically removed. We analyzed the EEG after displaying the stimulus 1000 ms later, where the baseline was 200 ms before the stimulus. Based on existing research results and the purpose of this study, we carried out statistical analyses on the amplitude and incubation period of N1, N2, N3, P2, and P3. This involved selecting the 12 electrode position, and carrying out four-factor repeated measure analysis of variance of 2 (batting route: straight line, oblique) × 2 (batting time: T0, T1) × 2 (subject type: tennis professional athletes, tennis second-grade athletes) × 4 electrode position (frontal area: F3, Fz, F4; frontal middle area: FC3, FCz, FC4; pre-occiput P3, Pz, P4; post-occiput: PO5, POZ, PO6). In addition, all *p*-values were corrected according to Green–Geisser.

## Results

### Operation Check Measure

#### Comparison of Technical Level and Exercise Years Between the Two Groups of Subjects

We compared the technical level and exercise years between the two groups of subjects (**Table 1**). As can be seen, tennis professional athletes and second-grade athletes show significant differences in the technical level and exercise years.

**Table 1:** Statistic table of technical level and exercise years of subjects.

Group	Excise years	Level
Professional athlete group	12.78 ± 0.69	7.2 ± 0.65
Second-grade athlete group	6.10 ± 0.66	3.87 ± 0.35
*t*-value	27.14	17.48
*p-*value	<0.001	<0.001


#### Comparison of Picture Familiarity

Comparison of the picture familiarity of the two groups of subjects at the T0, T1 time points (**Table 2**), shows that there is no significant difference in T0, T1picture familiarity between professional tennis athletes and second-grade athletes, and this can be used as experimental material for testing.

**Table 2:** Familiarity of pictures.

Group	T0	T1
Professional athlete group	2.68 ± 0.41	2.52 ± 0.37
Second-grade athlete group	2.77 ± 0.39	2.65 ± 0.38
*t*	1.12	1.38
*p*	>0.05	>0.05


### Behavior Results

#### Accuracy Rate

**FIGURE 2:**
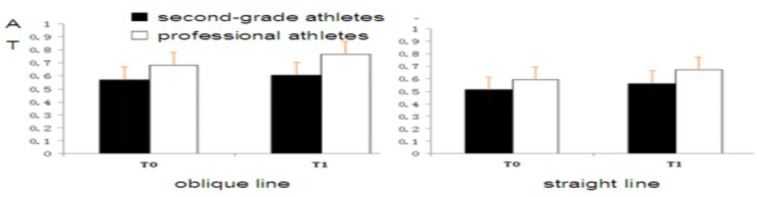
Accuracy rate of subjects of professional athletes and second-grade athletes at T0, T1.

A three-factor repeated measures analysis of variance of accuracy rates was conducted, and the results showed that the main effect of the batting route was significant [*F*(1,26) = 11.18, *p* < 0.001, $\eta_{p}^{2}$ = 0.21]. That is, the accuracy rate of subjects perceptual anticipation on an oblique line is significantly higher than on a straight line; thus, the main effect of the subject group was significant [*F*(1,26) = 18.5, *p* < 0.001, $\eta_{p}^{2}$ = 0.42]. The accuracy rate of professional athletes was significantly higher than that of the second-grade athletes and the main effect of time-points is significant [*F*(1,26) = 23.18, *p* < 0.001, $\eta_{p}^{2}$ = 0.21]. That is, the accuracy rate of T1 time points is significantly higher than the T0 time point (**Figure 2**).

The interaction between the batting route and the subject group was significant [*F*(1,26) = 14.68, *p* < 0.001, $\eta_{p}^{2}$ = 0.17]. The simple effect analysis found that the accuracy rate of professional athletes and the second-level athletes on the oblique batting line was significantly higher than for the straight line [all *F*(1,26) > 5.76, all *p* < 0.01]. The interaction between the time point and subject group was significant [*F*(1,26) = 11.28, *p* < 0.001, $\eta_{p}^{2}$ = 0.20]. The results of the simple effect analysis showed that the accuracy rate of professional athletes at T0 and T1 is significantly higher than that of second-grade athletes [all *F*(1,26) > 5.17, all *p* < 0.05]. Finally, the interaction between the time point and the batting rout was significant [*F*(1,26) = 8.97, *p* < 0.05, $\eta_{p}^{2}$ = 0.15]. The results of simple effect analysis showed that the accuracy rate of the oblique line at T0 and T1 is significantly higher than that of the straight line [all *F*(1,26) > 6.52, all *p* < 0.05].

#### Reaction Time

**FIGURE 3:**
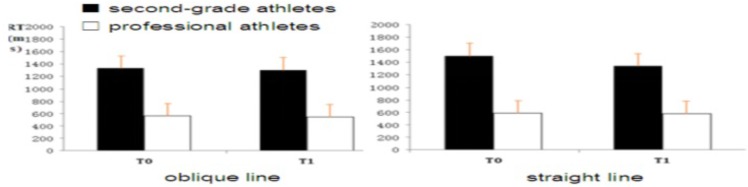
Reaction time of subjects of professional athletes and second-grade athletes at T0, T1.

Through the three-factor repeated measures analysis variance of reaction time, it was found that the main effect of the batting route was significant [*F*(1,26) = 30.12, *p* < 0.001, $\eta_{p}^{2}$ = 0.28], that is, the reaction time of subjects’ perceptual anticipation on the oblique line was significantly faster than on the straight line, and the main effect of the subject group was significant [*F*(1,26) = 55.75, *p* < 0.001, $\eta_{p}^{2}$ = 0.49]. Thus, the reaction time of professional athletes was significantly faster than that of second-grade athletes. The main effect of the time-point is significant [*F*(2,52) = 44.65, *p* < 0.001, $\eta_{p}^{2}$ = 0.43]: the reaction time of T1 time points is significantly faster than the T0 time point (**Figure 3**).

The interaction between the batting route and the subject group was significant [*F*(1,26) = 7.15, *p* < 0.001, $\eta_{p}^{2}$ = 0.15]. The simple effect analysis found that the reaction time of professional athletes and second-level athletes on the oblique batting line was significantly faster than on the straight line [all *F*(2,52)>5.06, all *p* < 0.01]. The interaction between the time point and subject group was significant [*F*(1,26) = 18.45, *p* < 0.001, $\eta_{p}^{2}$ = 0.21]. The results of the simple effect analysis showed that the reaction time of the professional athletes at T0 and T1 was significantly faster than that of the second-grade athletes [all *F*(1,26)>4.57, all *p* < 0.05]. The interaction between the time point and the batting route was significant [*F*(1,26) = 6.78, *p* < 0.05, $\eta_{p}^{2}$ = 0.10]. The results of simple effect analysis showed that the reaction time of the oblique line at T0 and T1 was significantly faster than that of the straight line [all *F*(1,26) > 6.54, all *p* < 0.05].

### ERP Results

**FIGURE 4:**
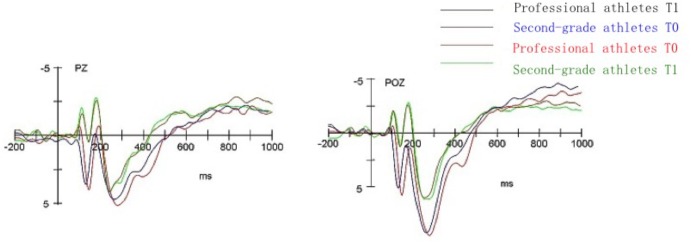
The mean ERP components wave of professional athletes and second-grade athletes straight judge at PZ, POZ electrode point at T0, T1 point of time.

**FIGURE 5:**
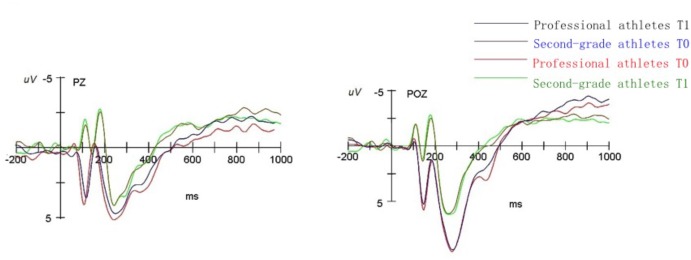
The mean ERP components wave of professional athletes and second-grade athletes oblique judge at PZ, POZ electrode point at T0, T1 point of time.

**Figures 4, 5** show the ERP total average map induced by two groups of subjects under the condition of two time points. The total average map of the parieto-occipital region (Pz, POz) showed that it induced significant negative components [N1 (80–120 ms), N2 (160–220 ms)] and significant positive components [P2 (120–160 ms) and P3 (220–370 ms)].

#### N1 (80–120 ms)

Through the four-factor repeated measures analysis variance between subjects of average amplitude of N1, it was found that the main effect of the subjects group is significant [*F*(1,26) = 7.35, *p* < 0.05, $\eta_{p}^{2}$ = 0.22]. The second-grade athlete group induced a larger N1 amplitude than the professional athletes group.

#### P2 (120–160 ms)

Through the four-factor repeated measures analysis variance between subjects of average amplitude of P2, it was found that the main effect of the subjects group was significant [*F*(1,26) = 9.62, *p* < 0.01, $\eta_{p}^{2}$ = 0.27]. The professional athletes group induced a larger P2 amplitude than the second-grade athlete group. The interaction edge of time point and electrode point was significant [*F*(1,26) = 3.37, *p* = 0.07, $\eta_{p}^{2}$ = 0.11], and the simple effect analysis showed that the electrode position in the pre-occipital region induced a larger P2 amplitude than in the post-occipital region at T0 and T1.

#### N2 (160–220 ms)

Through the four-factor repeated measures analysis of variance between the subjects of average amplitude of N2, it was found that the main effect of the subjects group is significant [*F*(1,26) = 4.22, *p* < 0.05, $\eta_{p}^{2}$ = 0.14]. The second-grade athlete group induced a larger N2 amplitude than the professional athletes group. The interaction of subject type and electrode point was significant [*F*(1,26) = 7.85, *p* < 0.05, $\eta_{p}^{2}$ = 0.23]. The simple effect analysis showed that the subjects of the second-grade athletes induced a larger N2 amplitude at the electrode in the frontal middle area than that of professional athletes. The interaction of batting route and electrode point was significant [*F*(1,26) = 3.11, *p* = 0.08, $\eta_{p}^{2}$ = 0.10]. The simple effect analysis also showed that the straight-line anticipation at the frontal middle area induced a larger N2 amplitude than the oblique line [*F*(1,26) = 3.41, *p* = 0.05, $\eta_{p}^{2}$ = 0.11]. Here, the simple effect analysis showed that the straight-line anticipation at the T2 time point induced a larger N2 amplitude than the oblique line.

#### P3 (220–370 ms)

Through the four-factor repeated measures analysis variance between subjects of average amplitude of P3 it was found that the main effect of the subjects group was significant [*F*(1,26) = 5.92, *p* < 0.05, $\eta_{p}^{2}$ = 0.18]. The professional athletes group induced a larger P3 amplitude than the second-grade athlete group.

#### Tracing Analysis Results

**FIGURE 6:**
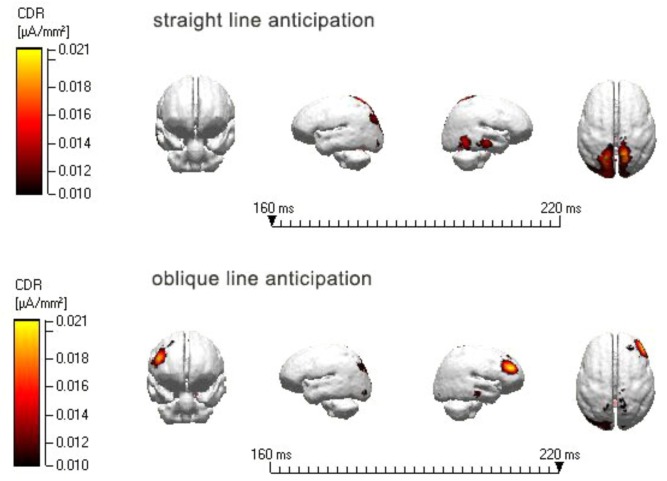
N2 tracing map of straight and oblique line anticipation of professional athletes at the T0, T1 time point.

Introduced the total average ERPs of different classes were into the Curry6.0 system with standard MRI heads, used SLORETA method to reconstruct the scalp activity of each task at different time in the three-shell spherical model. According to the ERPs components in experiment, carried out dipole localization analysis of N2 (160–220 ms) induced by the straight line and oblique line anticipation of professional athletes group at T1 time point. It was found that the N2 component induced by straight line anticipation at T1 time point significantly activated the superior parietal lobule at T1 time points, while the oblique line anticipation significantly activated the middle temporal gyrus (see **Figure 6**).

**FIGURE 7:**
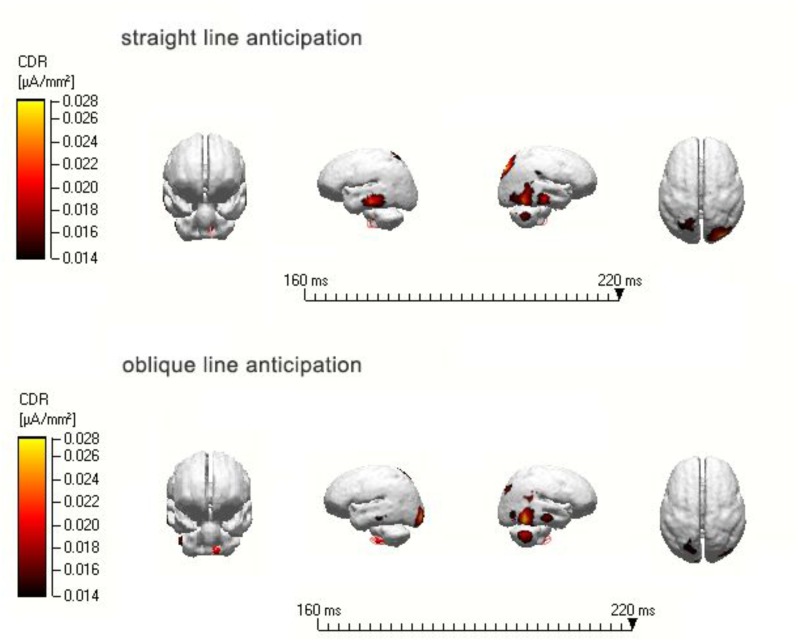
N2 tracing map of straight and oblique line anticipation of second-grade athletes at the T0, T1 time point.

Introduced the total average ERPs of different classes were into the Curry6.0 system with standard MRI heads, used SLORETA method to reconstruct the scalp activity of each task at different time in the three-shell spherical model. According to the ERPs components in experiment, carried out dipole localization analysis of N2 (160–220 ms) induced by the straight line and oblique line anticipation of second-grade athletes group at T0 time point. It was found that the N2 component induced by straight line anticipation at T0 time point significantly activated the culmenat T1 time point, while the oblique line anticipation significantly activated the middle frontal gyrus (see **Figure 7**).

## Discussion

### The Behavioral Advantage From Tennis Players’ Perceptual Anticipation

In this study, we investigated the differences in the cognitive processing of perceptual anticipation in tennis players at different levels before batting. The results show that the accuracy rate of professional athletes at T0 and T1 is significantly better than that of second - grade athletes. These findings are consistent with previous studies (Nakamoto and Mori, 2012; Zhang, 2012; Wang, 2013; Shang and Fan, 2015). Shang and Fan (2015) found that professional athletes and second-grade athletes appear to show differences on the perceptual anticipation of batting route at the T0, T1 time point, so this study also selected the T0 and T1 time points to examine the anticipation differences of professional athletes and second-grade athletes on batting routes at the two time points. Huys et al. (2009) and Farrow and Reid (2012) found that when it covers different parts of the opponent’s body, the information pickup method of skilled athletes is more global than that of low-skilled athletes when examining the anticipation ability of tennis players at different professional levels (Loffing et al., 2011; Smeeton and Huys, 2011). Loffing et al. (2011) studied the ability of high-level tennis players and novice athletes to anticipate the shot direction of the opponent after watching experiment materials delivered by different presentation methods (such as video presentation, spot presentation, and so on). As a result, the perceptual anticipation of the two groups of subjects on experimental materials with spot presentation were obviously worse than that with the video presentation, but the anticipation ability of the high-level tennis player was superior to the novice under all conditions (Loffing et al., 2011; Smeeton and Huys, 2011).

There are other studies that show that expert players can make better use of the early clues of sport events to generate more effective predictions of future events and receive significant behavioral advantage (Chi, 2012). Moreover, in those sports dominated by separate net class skills such as table tennis and badminton, the performance advantage of professional athletes from perceptual anticipation is also confirmed (Rose and Christina, 1990). Therefore, the results of these studies may show that in comparison with second-grade athletes, there is a set of complex and fine motion images in the brains of tennis professional athletes that allow match-ups with the actual situation, resulting in an overall advantage from perceptual anticipation. This overall advantage may also assist tennis professional athletes in high-level competitions, covering such decisions as how to make accurate and rapid response measures in the case of an opponent’s attack, the formation of behavioral habits, and the ability to make rapid decisions, and their responses will reflect what they have gained through repeated experience and reaction in competitive situations.

The results also show that the reaction time of professional athletes is significantly faster than the second-grade athletes at the T0 and T1 time points, which is consistent with previous studies (Nakamoto and Mori, 2012; Zhang, 2012; Wang, 2013; Shang and Fan, 2015). N’Diaye et al. (2004) have shown that the time identification and response speed of tennis experts was significantly faster than those of the experience group and novice groups. The response speed of the novice group was slowest, while the expert group had stronger attention and faster speed of concentration through long-term professional training. Mcpherson (2010) studied the perceptual anticipation ability of female high-level tennis players and found that the anticipation of the experts group on the batting route was significantly faster than that of the novice group. Therefore, the results of these studies show that compared with the second-grade athletes, tennis professional athletes have the ability to use existing stimulating information at high levels of efficiency and speed even where the stimulating information on display is not abundant.

### ERP Characteristics of Tennis Players’ Perceptual Anticipation

This study also examined the time course and spatial distribution characteristics of different levels of tennis players at the T0, T1 time points. The results of the ERP studies showed that in the early stage of processing the experimental material, second-grade athletes induced a higher negative N1 amplitude than the professional athletes. This result is similar to those of previous studies. Walsh and Kulikowski (1998) showed that long-term special training and perceptual learning gives athletes the ability to grasp the timing of attack and the best offensive point, and their perceptual skills have developed rapidly to improve the efficiency of information extraction. The study of Iguchi and Hashimoto (2000) shows that the early components of ERPs are the reflection of a number of different types of mental processes. N1 shows that in the early stages of perceptual cognitive processing the use of individual resources is generally interrelated (Iguchi and Hashimoto, 2000). The study of Zhao (2010) showed that the excellent Sanda athletes induced obvious N1 components in the central, central top, top, parieto-occipital regions, and in the occipital region of the cortex. There are also studies that show that when future events are unpredictable we may need to devote more attention and cognitive resources. Thus, these results may indicate that when the target stimulus appears, the second-grade athlete group begins to consume more attention resources in the early stage of cognition, resulting in the early consumption of their cognitive resources. This is found in the ERP component where the peak value of N1 component is far larger than in the professional athletes group.

Our results also show that the professional athletes induced a larger P2 amplitude than the second-grade athletes in a similar way to those of previous studies. Previous studies have shown that P2 components may allow quick detection of stimulating characteristics, and the sensitive stimulus will receive more attention (Karayanidis and Michie, 1996; Thorpe et al., 1996). In addition, previous studies have shown that the stimulus with stronger biological importance is more noticeable and can elicit larger P2 amplitudes (Carretié et al., 2001). It has also been found that P2 components are time-related components. The study of Gontier et al. (2008) also observed significant positive components around 200 ms after the time interval, and they thought that P2 represents the characterization of working memory. Thus, the results of these studies may indicate that the subjects in different levels have different degrees of cognitive processing in the time perception process. Professional tennis athletes induced the biggest P2 amplitude, which is probably because they have a higher amount of their working memory invested in the process of time perception compared with second-grade athletes. The observation ability and alertness of professional athletes have also been greatly improved, which means that they can sense stimuli early and carry out control and processing from their years of tennis professional training and high-level athletic experience.

The results also demonstrated that the N2 amplitude elicited by the second-grade athletes was larger than the professional athletes group. This is also consistent with previous studies. Previous studies have shown that the N2 component mainly reflects the psychological processing process of identifying the characteristics of the target stimulus, and the larger N2 amplitude may indicate self-regulation of the reaction induced by a stimulus in continuous attention control and perceptual exercises. Studies have found that N2 usually reflects the judgment of stimulus in lateral vision, and the amplitude is significantly adjusted by the scope of attention (Posner and Gilbert, 1999). In recent years, N2 has often been used to reflect stimulus identification, attention mobility (Patel and Azzam, 2005), reaction suppression, conflict monitoring (Azizian et al., 2006), novice, or matching detection (Folstein and Van Petten, 2008). In this study, since N2 follows the P2 component, it may more closely represent the evaluation of the cognitive and matching degree of information.

Tennis professional athletes have rich experience in tennis training and competition. When the stimulus matches the experience memory, their identification technique characteristics and the degree of matching recognition of them are higher than that of second-grade athletes. As the second-grade athletes have less training and competition experience than professional athletes, although their brain also forms a certain special knowledge system, the degree of consolidation is inferior to that of tennis professional athletes. Thus, the evaluation effect after the matching cognition is also not as good as that of professional athletes.

The results of the present study show that professional athletes induce a larger P3 amplitude than second-grade athletes. These results are also similar to those of previous studies. Previous studies have shown that the P3 component is the posterior component generated in the brain’s nerve center after individual sense the stimulus information, which reflects the speed of brain for identification, classification, and encodes of extraneous stimulus; in other words, it reflects the required time of evaluation (Kok, 2001; Dockree et al., 2005; Kimura et al., 2005). After the presentation of a stimulus, the subjects were able to selectively pay attention to the information related to the target stimulus, and concentrate their main attention on the required stimulus-sensing materials and the pattern resolution, combining with the field-sensing and memory process to retrieve the brain’s long-term memory structure and extract the appropriate stimulus information to match them (Kok, 2001; Dockree et al., 2005; Kimura et al., 2005).

Jin et al. (2011) recorded, analyzed, and compared the behavior data and ERP data of professional badminton players and non-professional badminton players watching different levels of badminton. This study concluded that professional players have more accurate judgments of the placement of badminton strokes than non-professional players, showing good anticipation ability. Zhou and Feng (2011) found that when fleuret players of different levels identify technical movements and spatial perception in the static context, the appeared time of P3 induced in the specific brain area is different. Zhang (2012) argued that the level of athletes and the processing speed of stimulus material were positively correlated. In addition, Jin et al. (2011), Zhou and Feng (2011), and other studies have declared that the P3 amplitude of professional athletes is significantly larger than that of non-professional athletes.

Dunchin (1981) proposed the “context updating model” theory in this context, and they believe that environmental information is stored in the brain’s information base in the form of characterization after it is accepted, becoming the contents of the memory. When new stimulus information is accepted, the new stimulus will be affected by related old characterizations when it is in cognitive processing and will be integrated into the original characterization to form a new characterization. All characterization related to the information is called context. Thus, the cognitive processing of new information is used to amend the original context, and the size of the amplitude represents the amount of context correction. Therefore, the results of P3 in this study may indicate that the subjects’ brain conducted superior amendments of knowledge characterization in the later stages of visual searching of spatial contexts, completed the judgment and identification of the best offensive point. P3 amplitude also reflects the individual’s amendment of the background. The larger the background need to amend, the larger the P3 amplitude. Therefore, these results may indicate that professional athletes who have accumulated a lot of tennis-related knowledge and skills have a strong ability to amend the background in different competition environments, and their P3 amplitude is therefore large, while the second-grade athletes group have a certain degree of amendment ability of the background, but because the tennis competitions they participate in are lower, their competitive experience is not strong. Thus, once they encounter complex sports scenes, they cannot carry out amendments, so the results showed a small amplitude. In addition, the subjects of the second-grade athlete group induced a larger N2 amplitude on the middle electrode of the frontal middle region than the professional athlete. This is again similar to previous studies. Previous studies have shown that the N2 effect in the frontal region of brain is associated with perceptual conflict, and N2 induces a larger amplitude in the case of conflict than in the absence of conflict (Chen et al., 2010; Yuan et al., 2010; Van Veen and Carter, 2015). Therefore, these results may indicate that the frontal region reflects conflict in perceptual anticipation, which is the key area for an athlete when carrying out perceptual anticipation.

Furthermore, this study also used a dipole tracing technique to carry out dipole location analysis of N2 (160–220 ms) induced by the straight line and oblique line of experts at the T1 time point. It was found that the N2 component induced by straight line anticipation at the T1 time point was significantly activated in the superior parietal lobule, while the oblique line anticipation activated the temporal gyrus through dipole location analysis of N2 (160–220 ms) induced by straight line and oblique lines of experts at the T0 time point. It was found that the N2 component induced by straight line anticipation at the T0 time point significantly activated the culmen, while the oblique line anticipation activated the middle frontal gyrus.

Northoff et al. (2004) found that the frontal region is the most important node in the sport-related brain network through fMRI technology. Milton et al. (2007) studied the brain patterns associated with the activity expectation of golfing experts and non- athletes in the process of preparing to shoot. The results show that golfing experts are mainly activated in the frontal, superior parietal, and occipital lobes. Davis et al. (2014) found similar results to Milton et al. (2007). Previous studies have also found that the prefrontal cortex of brain is involved in information processing related to perception (Singer et al., 1996). Therefore, these findings suggest that the prefrontal cortex may be a critical area of perceptual anticipation in tennis, and is responsible for coordinating the conflict of perceptual anticipation and determining the efficiency of anticipation.

## Conclusion

Compared with the second-grade athletes group, professional tennis athletes have significant behavioral advantages in the anticipation of batting route, both before the time of batting 80 ms ago (T0) and the landed time of the ball (T1). ERP results showed that the second-grade athletes induced larger N1 and N2 amplitudes than the professional group. The straight-line anticipation in the electrode position of the frontal region induced a larger N2 amplitude than the oblique line anticipation, while at the T0 and T1 time points, straight-line anticipation induced a larger N2 amplitude than the oblique line anticipation, and the professional group induced larger P2 and P3 amplitudes than the second-grade athletes group. Meanwhile, at the T0, T1 time points, the expert group showed faster and more accurate decision-making, invested less brain resources into the perception stage of the stimulation task, but allocated a large amount of cognitive resources to the type and processing stage of stimulus materials. The processing stage investment enabled them to quickly extract and integrate information, giving a clear cognitive advantage. The prefrontal cortex of the human brain may therefore be a critical area for perceptual anticipation in tennis.

## Ethics Statement

The Ethics Committee of Hunan Normal University approved this study.

## Author Contributions

RS designed the experiment and wrote the paper. RS and YC conducted the experiment. YC collected the data.

## Conflict of Interest Statement

The authors declare that the research was conducted in the absence of any commercial or financial relationships that could be construed as a potential conflict of interest. The handling Editor declared a shared affiliation, though no other collaboration with the authors.
